# Dose Adjustment of JAK Inhibitors for the Management of Moderate-to-Severe Atopic Dermatitis: A Single-Centre Retrospective Study

**DOI:** 10.3390/medicina62061127

**Published:** 2026-06-09

**Authors:** Costanza Falcidia, Francesco D’Oria, Giulio Foggi, Paola Facheris, Matteo Bianco, Luciano Ibba, Alessandra Narcisi, Antonio Costanzo, Luigi Gargiulo

**Affiliations:** 1 Dermatology Unit, IRCCS Humanitas Research Hospital, 20089 Rozzano, Italy; costanza.falcidia@humanitas.it (C.F.); francesco.doria@humanitas.it (F.D.); giulio.foggi@humanitas.it (G.F.); paola.facheris91@gmail.com (P.F.); matteo.bianco@humanitas.it (M.B.); luciano.ibba@humanitas.it (L.I.); alessandra.narcisi@hunimed.eu (A.N.); antonio.costanzo@hunimed.eu (A.C.); 2 Department of Biomedical Sciences, Humanitas University, 20072 Pieve Emanuele, Italy; 3 Dermatology Unit, Sant’Anna Hospital, ASST-Lariana, 22100 Como, Italy

**Keywords:** JAK-inhibitors, abrocitinib, upadacitinib, dose-adjustment, atopic dermatitis

## Abstract

*Background and Objectives*: Janus kinase (JAK) inhibitors have expanded the therapeutic options for moderate-to-severe atopic dermatitis (AD). The possibility of dose modulation with abrocitinib (100 and 200 mg) and upadacitinib (15 and 30 mg), both selective JAK1 inhibitors, represents a potential clinical advantage, allowing dose escalation in cases of insufficient response and dose de-escalation in the setting of poor tolerability or during maintenance treatment. However, real-world data remain limited, and the rationale for dose adjustment is not always standardized. This study aimed to describe, using a real-world database, the frequency, timing, and reasons for dose modifications of these agents. *Materials and Methods*: We retrospectively analyzed the clinical data of 212 patients with moderate-to-severe AD treated with abrocitinib (n = 47) or upadacitinib (n = 165). Dose adjustments, including dose escalation and dose de-escalation, were recorded together with their timing and clinical reasons. *Results*: In the abrocitinib group, 34/47 patients (72.3%) initiated treatment at 100 mg, whereas 13/47 patients (27.7%) started at 200 mg. Six patients (12.8%) underwent a dose adjustment. One patient (2.1%) switched from 200 to 100 mg because of complete AD remission and concomitant menstrual cycle alterations, whereas five patients (10.6%) underwent dose escalation from 100 to 200 mg because of incomplete disease control. Among the six abrocitinib-treated patients who underwent dose adjustment, achievement of IGA 0/1 after dose modification was documented in all cases. In the upadacitinib group, 93/165 patients (56.4%) started at 15 mg, whereas 72/165 patients (43.6%) started at 30 mg. Overall, 44/165 patients (26.7%) underwent at least one dose adjustment, accounting for a total of 50 dose modifications: 27 escalations from 15 to 30 mg and 23 de-escalations from 30 to 15 mg. Among patients initiating treatment at 15 mg, 23/93 patients (24.7%) increased the dose to 30 mg after a median of 33.1 weeks because of suboptimal disease control. Among those starting at 30 mg, 21/72 patients (29.2%) reduced the dose to 15 mg after a median of 44.3 weeks. Of these, 12/21 patients (57.1%) reduced the dose because of adverse events, including herpetic infections and acne, whereas the remaining patients de-escalated because of optimal disease control. Some patients underwent multiple dose modifications: four followed a 30→15→30 mg sequence, with re-escalation after 13.2 weeks because of suboptimal disease control, and two followed a 15→30→15 mg sequence, with dose reduction after approximately 26.7 weeks because of herpes zoster. Overall, 29/44 patients achieved IGA 0/1 within 16 weeks and 38/44 within 32 weeks after dose modification. *Conclusions*: In this real-world cohort, dose adjustments of selective JAK1 inhibitors were frequently performed in patients with moderate-to-severe AD, particularly among those treated with upadacitinib. Dose escalation was mainly used to address suboptimal disease control, whereas dose de-escalation was performed in the setting of adverse events or optimal disease control. The availability of two dosing regimens may allow treatment intensity to be adapted to individual disease severity, response, and tolerability, supporting a personalized approach to AD management.

## 1. Introduction

Atopic dermatitis (AD) is a chronic, relapsing inflammatory skin disease characterized by epidermal barrier dysfunction, immune dysregulation, intense pruritus, sleep disturbance, and a substantial impact on quality of life, particularly in patients with moderate-to-severe disease [1]. In recent years, the Janus kinase/signal transducer and activator of transcription (JAK–STAT) pathway has emerged as a key therapeutic target in AD, as several cytokines involved in disease pathogenesis and itch, including IL-4, IL-13, IL-22, IL-31, thymic stromal lymphopoietin, and interferon-γ, signal through JAK-dependent pathways [2]. Among JAK family members, JAK1 has a central role in type 2 inflammation, pruritus amplification, keratinocyte dysfunction, and impaired skin barrier integrity [3,4].

This biological rationale has supported the development of selective JAK1 inhibitors for moderate-to-severe AD. Abrocitinib and upadacitinib have demonstrated rapid and clinically meaningful efficacy in pivotal phase III clinical trials, leading to the approval of two dosing regimens for each drug: abrocitinib 100 and 200 mg, and upadacitinib 15 and 30 mg [5,6,7,8]. Taken together, these trials established selective JAK1 inhibition as an effective systemic strategy in AD and provided the basis for the two dose levels currently available in clinical practice.

Building on this fixed-dose evidence, the phase IIIb/IV Flex Up study specifically addressed whether upadacitinib treatment could be adapted according to clinical response. In this treat-to-target trial, patients receiving upadacitinib 15 mg who had not achieved EASI 90 after 12 weeks were escalated to 30 mg, whereas those receiving 30 mg who had achieved EASI 90 were reduced to 15 mg. By week 24, 48.1% of patients undergoing escalation achieved EASI 90, while 68.5% of those undergoing dose reduction maintained EASI 90; improvements in pruritus were also observed, and no new safety signals emerged [9]. These findings are particularly relevant because they suggest that dose adjustment may be used not only to improve inadequate response, but also to maintain disease control with a lower treatment intensity once a stringent target has been achieved.

Despite this rationale, evidence on dose adjustment of JAK1 inhibitors in routine clinical practice is still limited. For upadacitinib, available data are mainly restricted to the Flex Up trial and a recent multicenter real-world study, both suggesting that dose escalation and de-escalation may be clinically useful according to response, achievement of treatment targets, or tolerability [9,10]. By contrast, for abrocitinib, the published literature has primarily focused on the efficacy and safety of the approved dose regimens [11,12], whereas real-world information on how dosing is modified over time in daily practice remains scarce. This gap is clinically relevant because the long-term management of atopic dermatitis often requires flexible and individualized treatment decisions that are not fully captured by fixed-dose trial designs.

The aim of our study was to investigate, in a real-world monocentric cohort of patients with moderate-to-severe AD, the frequency, timing, and reasons for dose adjustment of abrocitinib and upadacitinib in routine clinical practice, thereby providing practical data on the role of dose flexibility in the long-term management of AD.

## 2. Materials and Methods

We performed a retrospective, monocentric, real-world study based on a clinical database developed at the Dermatology Unit of IRCCS Humanitas Research Hospital, Rozzano (Milan), Italy. The database included patients with moderate-to-severe atopic dermatitis seen in routine clinical practice over a 2-year period, from January 2023 to December 2025. Adult patients receiving the selective JAK1 inhibitors abrocitinib or upadacitinib were included in the present analysis. Abrocitinib was prescribed at the approved doses of 100 mg or 200 mg once daily, and upadacitinib at 15 mg or 30 mg once daily, according to routine clinical judgment and the approved Summary of Product Characteristics of both drugs [13,14]. The aim of the study was to describe the frequency, timing, and clinical reasons for dose adjustment, as well as the main escalation and de-escalation patterns observed in daily practice. Clinical and demographic data were retrospectively collected from the database, including age, sex, baseline treatment dose, occurrence and type of dose modification, reason for dose adjustment, and timing of dose changes. Dose adjustment was defined as any switch between the two approved dose levels of the same drug during follow-up. Patients undergoing more than one dose modification were recorded as having multiple dose adjustments, and these were also considered at the event level when appropriate. In addition, disease severity was assessed through Eczema Area and Severity Index (EASI) values before and after dose modification, in order to explore the clinical course associated with dose adjustment. Because of the retrospective real-world design, post-adjustment follow-up visits were not scheduled at predefined timepoints. Therefore, the first post-adjustment EASI assessment was defined as the first available EASI value recorded after dose modification for each patient. Overall, 212 patients with moderate-to-severe atopic dermatitis were included, of whom 47 were treated with abrocitinib and 165 with upadacitinib. Data analysis was descriptive and was performed using Microsoft Excel (Microsoft Corp., Redmond, WA, USA). Categorical variables were reported as absolute numbers and percentages, whereas continuous variables were summarized as mean ± standard deviation or median and interquartile range, as appropriate. For key proportions, two-sided 95% confidence intervals were calculated using the exact binomial method, in order to provide an estimate of precision around descriptive outcomes.

Time to dose adjustment was summarized descriptively using median, interquartile range, and range. Time-to-event analyses were not performed because dose adjustment was considered a heterogeneous clinical management decision rather than a single irreversible outcome, as modifications could occur in different directions and for different reasons, including insufficient disease control, adverse events, or remission.

The study was conducted in accordance with the principles of the Declaration of Helsinki. All patients had provided written informed consent for the retrospective analysis of clinical data collected during routine practice.

## 3. Results

### 3.1. Study Population

This monocentric retrospective study included 212 patients with moderate-to-severe atopic dermatitis treated with selective JAK1 inhibitors in routine clinical practice, of whom 47 received abrocitinib and 165 upadacitinib. In the abrocitinib cohort, 34/47 patients (72.3%) started treatment with 100 mg and 13/47 (27.7%) with 200 mg. In the upadacitinib cohort, 93/165 patients (56.4%) started with 15 mg and 72/165 (43.6%) with 30 mg. Median overall follow-up was 52 weeks (IQR 16–52) in the abrocitinib cohort and 52 weeks (IQR 16–104) in the upadacitinib cohort.

### 3.2. Abrocitinib Dose-Adjustment Outcomes

Among the 47 patients with moderate-to-severe atopic dermatitis treated with abrocitinib, 23 (48.9%) were male and 24 (51.1%) were female. Mean age was 39.5 ± 16.1 years, and the mean baseline EASI score was 20.58. Overall, 34 patients (72.3%) started abrocitinib 100 mg, whereas 13 (27.7%) started abrocitinib 200 mg. Dose adjustment was observed in six patients (12.8%; 95% CI: 4.8–25.7%), and their characteristics are shown in Table 1. Complete demographic data of our cohort treated with abrocitinib are shown in Table 2. In particular, one patient (2.1%; 95% CI: 0.1–11.3%) underwent dose reduction from abrocitinib 200 mg to 100 mg after 13 weeks of treatment because of optimal control of AD (defined by EASI ≤ 3 and/or IGA 0/1), and concomitant menstrual cycle alterations that resolved after dose reduction and never reoccurred. The EASI remained 0 until the latest follow-up, which occurred at week 104. By contrast, five patients (10.6%; 95% CI: 3.5–23.1%) underwent dose escalation from 100 mg to 200 mg because of incomplete disease control. The median time to dose adjustment was 8.0 weeks (IQR 4.4–9.9; range 0.6–13.4). Among these latter patients, the mean absolute EASI at the time of dose switching was 7.0. The first post-switch follow-up assessment was performed after a mean of 34 weeks, at which time, the mean EASI was 0 in 4/5 patients. This favorable response was maintained through the last available follow-up recorded at week 52 for 5/6 patients with a mean EASI of 0. However, for 1/6 patients, we recorded an EASI of 0.2 at week 104. Achievement of IGA 0/1 after dose adjustment was documented in all six dose-adjusted patients. The mean time to IGA 0/1 was 19.6 weeks, with a median of 16 weeks (IQR 13.4–16; range 4–52). Overall, 5/6 patients achieved IGA 0/1 within 16 weeks after dose modification, while one patient achieved IGA 0/1 at week 52.

No adverse events were reported.

### 3.3. Upadacitinib Dose-Adjustments Outcomes

Among the 165 patients treated with upadacitinib, 97 (58.8%) were male and 68 (41.2%) were female. Mean age was 41.9 ± 17.1 years, and the mean baseline EASI score was 16.44. Overall, 93/165 patients (56.4%) started upadacitinib 15 mg, whereas 72/165 (43.6%) started upadacitinib 30 mg. Dose adjustment was observed in 44/165 patients (26.7%; 95% CI: 20.1–34.1%), accounting for a total of 50 dose-change events, including 27 escalations from 15 mg to 30 mg and 23 de-escalations from 30 mg to 15 mg (Table 3 and Table 4).

Among patients starting with 15 mg, 23/93 (24.7%; 95% CI: 16.4–34.8%) increased the dose to 30 mg after a median of 33.1 weeks (IQR 19.8–78.5) because of suboptimal disease control. The mean absolute EASI at the time of dose adjustment was 9.1. The first post-switch follow-up assessment was performed after a mean of 18.6 weeks, at which time the mean absolute EASI was 4.65. The mean absolute EASI was 2.0 at the latest follow-up, which occurred at a median of 52 weeks (IQR 52–52; range 16–52).

Among those starting with 30 mg, 21/72 (29.2%; 95% CI: 19.0–41.1%) reduced the dose to 15 mg after a median of 44.3 weeks (IQR 23.1–92.7). Of the latter, 12/21 (57.1%; 95% CI: 34.0–78.2%) underwent dose reduction because of adverse events, including herpes zoster, acne, herpes simplex, weight gain, and nausea. These patients achieved sustained resolution of adverse events after dose reduction, without recurrence during the latest follow-up.

Whereas the remaining 9/21 (42.9%; 95% CI: 21.8–66.0%) patients reduced the dose after achieving optimal disease control, defined by EASI< or =3 and/or IGA 0/1. Indeed, the mean absolute EASI at the time of dose de-escalation was 2.0. This favorable response was maintained throughout the last available follow-up recorded after a median of 52 weeks (IQR 25–104; range 16–156), in which the mean absolute EASI was 1.05.

Multiple dose adjustments were also observed, with 4 patients showing a 30→15→30 sequence and 2 patients a 15→30→15 sequence. In the patients with a 30→15→30 sequence, re-escalation occurred after a median of 13.2 weeks from dose reduction because of inadequate disease control with upadacitinib 15 mg. In the two patients who underwent a 15→30→15, dose de-escalation occurred after 26.7 weeks from the escalation for both; in one patient, it was associated with herpes zoster after escalation, whereas in the other, it followed remission. The mean time to achieve IGA 0/1 was 22.7 weeks, with a median of 16 weeks (IQR 16–32; range 8–52). Overall, 29/44 patients achieved IGA 0/1 within 16 weeks and 38/44 within 32 weeks after dose modification.

Baseline characteristics according to dose-adjustment status are summarized in Table 5. Baseline IGA values did not show a pattern of greater disease severity among patients who subsequently underwent dose adjustment. In the abrocitinib cohort, mean baseline IGA was similar between dose-adjusted and non-dose-adjusted patients, whereas in the upadacitinib cohort, it was numerically lower among dose-adjusted patients. Similarly, baseline EASI values, when available, did not suggest a consistent trend toward higher baseline severity in patients requiring subsequent dose modification. A possible explanation is that patients with lower baseline severity may have been more frequently started on the lower approved dose and subsequently required escalation in case of incomplete disease control, whereas patients with higher baseline severity may have been started directly on the higher dosage. Overall, these findings suggest that dose adjustment in routine clinical practice was not driven by baseline severity alone, but rather reflected a dynamic decision-making process influenced by initial dose selection, subsequent treatment response, achievement or maintenance of disease control, adverse events, and tolerability.

## 4. Discussion

This real-world monocentric study provides further evidence that dose adjustment is a relevant feature of the management of AD in patients with moderate-to-severe atopic dermatitis with JAK1 inhibitors in routine clinical practice. Overall, dose flexibility was observed with both upadacitinib and abrocitinib, although with different frequencies and patterns. In the upadacitinib cohort, 44 of 165 patients underwent at least one dose adjustment, accounting for 50 dose-change events, whereas in the abrocitinib cohort, dose adjustment was observed in 6 of 47 patients. Taken together, these findings suggest that dose modulation is not an exceptional occurrence in daily practice, but rather a pragmatic strategy used to adapt treatment intensity according to clinical response and tolerability over time. This observation is consistent with emerging dose-stratified real-world evidence on selective JAK1 inhibitors in AD [15].

The pattern of dose adjustment differed between the two JAK1 inhibitors. In patients treated with abrocitinib, dose changes were mainly represented by escalation from 100 mg to 200 mg because of incomplete disease control, whereas de-escalation from 200 mg to 100 mg was documented in only one patient after complete remission and concomitant onset of an AEs. In the latter patient, complete remission was maintained until the latest follow-up. By contrast, the upadacitinib cohort showed a broader and more dynamic spectrum of dose modification, including both escalation and de-escalation as well as multiple sequential adjustments. In particular, among patients starting with 15 mg, nearly one quarter subsequently increased the dose to 30 mg because of incomplete disease control, while among those starting with 30 mg, approximately one third reduced the dose to 15 mg, mainly because of AEs or complete remission of AD.

Our findings are broadly in line with the treat-to-target rationale explored in the phase IIIb/IV Flex Up study 33 [9], in which patients not achieving EASI90 after 12 weeks on upadacitinib 15 mg were escalated to 30 mg, whereas those achieving EASI90 on 30 mg were reduced to 15 mg. That study provided an important proof of concept that both escalation and de-escalation may be clinically meaningful within the same therapeutic strategy. Similarly, in our real-world cohort, escalation from 15 mg to 30 mg was consistently driven by inadequate disease control, whereas de-escalation from 30 mg to 15 mg reflected either the occurrence of adverse events or the achievement of satisfactory disease control defined by an absolute EASI score ≤ 3 or IGA 0/1 and in line with the AHEAD expert consensus recommendations [16]. Our results are also consistent with the limited but growing real-world literature specifically addressing upadacitinib dose flexibility. In a recent multicentre real-world study [10], dose escalation from 15 mg to 30 mg was reported in patients with insufficient clinical control, whereas dose reduction from 30 mg to 15 mg was mainly performed because of AEs or achievement of minimal disease activity. For the latter patients, minimal disease activity was maintained until the latest follow-up visit without clinically relevant relapses. In our cohort, a comparable overall treatment pattern emerged. Importantly, our study further expands currently available evidence by providing a patient-level description of multiple switch patterns, including 30→15→30 and 15→30→15 sequences. These cases underline that dose adjustment should not necessarily be viewed as a one-time decision but may represent a dynamic process during long-term treatment. The abrocitinib data are also noteworthy, particularly because real-world evidence on dose adjustment with this drug remains very limited.

The abrocitinib findings should be interpreted with caution. In this subgroup, the observed dose modifications were mostly represented by escalation from 100 mg to 200 mg for incomplete disease control. A possible interpretation is that, in our real-world setting, abrocitinib dose adjustment was mainly used as an intensification strategy in patients initially treated with the lower dose and showing suboptimal disease control. This pattern may reflect the greater efficacy generally associated with abrocitinib 200 mg compared with 100 mg, as consistently shown in phase 3 clinical trials [5,6].

From a practical standpoint, this flexible approach may also have relevant implications for treatment persistence. By offering an intermediate option between maintaining the same regimen unchanged and discontinuing or switching treatment altogether, dose adjustment may favor drug survival and prolong the usefulness of a given JAK1 inhibitor in routine care. In other words, dose modulation may help clinicians manage partial response or tolerability issues without immediately abandoning an otherwise effective mechanism of action. This aspect is particularly valuable in chronic diseases such as AD, in which long-term stability is often a major therapeutic goal. Another relevant implication is the possibility of intervening “within class,” thereby avoiding premature switches to different pharmacological classes. Moreover, in some patients, optimizing the dose of the same JAK1 inhibitor may reduce the need for early transition to another systemic treatment, potentially limiting instability during therapeutic transitions and preserving alternative options for subsequent lines of therapy. From this perspective, dose adjustment may represent not only a response-oriented strategy but also a way to preserve flexibility in the broader long-term therapeutic sequencing of moderate-to-severe atopic dermatitis.

From a safety perspective, AEs represented a major driver of dose reduction. The most frequently reported events leading to de-escalation included acne (n = 5), weight gain (n = 4), herpes zoster (n = 3), herpes simplex (n = 2), nausea (n = 1), and menstrual cycle alterations (n = 1). These findings are broadly consistent with the known tolerability profile of JAK1 inhibitors in both clinical trials and real-world studies [11,17,18,19,20].

In our cohort, menstrual cycle alteration was observed in one patient treated with abrocitinib 200 mg and resolved after dose reduction to 100 mg. Although causality cannot be established from a single case, this observation is noteworthy in light of recent real-world evidence describing menstrual cycle alterations in female patients treated with JAK inhibitors for dermatological diseases. The proposed biological rationale involves potential interference with the hypothalamic–pituitary–ovarian axis through leptin-related JAK2/STAT3 signaling. However, evidence specifically related to abrocitinib remains limited; therefore, our finding should be interpreted as an anecdotal real-world observation and a potential pharmacovigilance signal requiring confirmation in larger studies [21].

Notably, rather than leading directly to treatment discontinuation, these adverse events prompted dose reduction in a substantial proportion of patients. Indeed, in our study cohort, patients who underwent dose reduction because of adverse events experienced resolution of these events after de-escalation, with no recurrence reported during the available follow-up, suggesting that de-escalation may serve as a useful strategy to preserve treatment continuation. In this context, dose flexibility may be clinically advantageous not only for maximizing response but also for maintaining treatment persistence.

## 5. Conclusions

Our study has some limitations. First, its retrospective monocentric design may have introduced selection bias and limited the generalizability of the findings. Secondly, the study was descriptive in nature and was not designed to compare the two drugs formally or to identify predictors of dose adjustment. A further limitation is the marked imbalance between treatment cohorts, with a substantially smaller number of patients receiving abrocitinib than upadacitinib. Consequently, abrocitinib-related findings should be interpreted as descriptive and hypothesis-generating, rather than as conclusive evidence of drug-specific dose-adjustment patterns. Despite these limitations, this study offers a clinically relevant picture of how dose adjustment of oral JAK1 inhibitors is implemented in everyday dermatology practice. Our data suggest that dose flexibility is an important and pragmatic component of long-term treatment, allowing escalation in patients with inadequate disease control and de-escalation in patients with either adverse events or satisfactory disease control. Larger prospective real-world studies are needed to better define which patients are most likely to benefit from escalation or de-escalation strategies and to establish more standardized criteria for dose optimization over time.

## Figures and Tables

**Table 1:** Clinical characteristics of patients who underwent dose modification of abrocitinib.

Patient No.	Age, Years	Sex	Dose Adjustment	Reason for Adjustment	Dose Adjustment Timing (Weeks)	Achievement of IGA 0/1 (Weeks from Adjustment)
**1**	30, 5	F	200→100	Complete remission + menstrual cycle alteration	**13, 4**	13, 4
**2**	63, 4	M	100→200	Incomplete disease control	**4, 4**	16
**3**	53, 1	F	100→200	Incomplete disease control	**7, 6**	16
**4**	23, 3	M	100→200	Incomplete disease control	**9, 9**	52
**5**	19, 6	F	100→200	Incomplete disease control	**0, 6**	4
**6**	32, 3	M	100→200	Incomplete disease control	**8, 4**	16

**Table 2:** Demographic characteristics of the patients treated with abrocitinib in our study. * Baseline EASI was available for 27 patients.

Variable	Value
Patients treated with abrocitinib, n	47
Male, n (%)	23 (48.9)
Female, n (%)	24 (51.1)
Mean age, years ± SD	39.5 ± 16.1
Mean baseline EASI *	20.58
Initial dose 100 mg, n (%)	34 (72.3)
Initial dose 200 mg, n (%)	13 (27.7)
Patients with ≥1 dose adjustment, n (%)	6 (12.8)
Total dose-adjustment events, n	6
Escalation 100→200 mg, n (%)	5 (10.6)
De-escalation 200→100 mg, n (%)	1 (2.1)
Main reason for escalation	Suboptimal disease control
Reason for de-escalation	Complete remission and menstrual cycle alterations
Median time to dose adjustment, weeks (IQR)	8.0 (4.4–9.9)
Range, weeks	0.6–13.4

**Table 3:** Clinical characteristics of patients who underwent dose modification of upadacitinib.

Patient No.	Age, Years	Sex	Dose-Adjustment Pattern	Reason for Adjustment	Dose Adjustment Timing from Treatment Initiation, Weeks	Achievement of IGA 0/1 (Weeks from Adjustment)
1	47.0	Female	15→30	Incomplete disease control	8	16
2	24.0	Male	15→30	Incomplete disease control	50	16
3	24.0	Male	15→30	Incomplete disease control	32	16
4	42.0	Female	15→30	Incomplete disease control	145	16
5	73.0	Female	15→30	Incomplete disease control	19	32
6	27.0	Male	15→30	Incomplete disease control	34	52
7	51.0	Male	15→30	Incomplete disease control	87	52
8	50.0	Female	15→30	Incomplete disease control	56	16
9	41.0	Male	15→30	Incomplete disease control	18	52
10	34.0	Female	15→30	Incomplete disease control	16	32
11	23.0	Male	15→30	Incomplete disease control	78	52
12	34.0	Female	15→30	Incomplete disease control	80	32
13	21.0	Female	15→30	Incomplete disease control	128	16
14	56.0	Female	15→30	Incomplete disease control	76	52
15	29.0	Male	15→30	Incomplete disease control	20	8
16	19.0	Male	15→30	Incomplete disease control	64	32
17	48.0	Female	15→30	Incomplete disease control	39	8
18	37.0	Male	15→30	Incomplete disease control	72	16
19	45.0	Male	15→30	Incomplete disease control	10	16
20	38.0	Male	15→30	Incomplete disease control	25	16
21	24.0	Female	15→30	Incomplete disease control	27	16
22	24.0	Male	30→15	Complete remission	45	45
23	33.0	Male	30→15	AE: acne	70	16
24	42.0	Female	30→15	AE: acne; herpes simplex	40	16
25	41.0	Male	30→15	AE: weight gain	148	16
26	55.0	Male	30→15→30	Remission; incomplete disease control	23; 35	8; 16
27	17.0	Male	30→15→30	Remission; incomplete disease control	16; 30	8; 16
28	60.0	Male	30→15→30	Remission; incomplete disease control	56; 72	32; 8
29	45.0	Male	30→15→30	Remission; incomplete disease control	71; 79	16; 8
30	54.0	Female	15→30→15	Incomplete disease control; AE: herpes zoster	20; 46.7	32
31	52.0	Male	15→30→15	Incomplete disease control; remission	19.9; 46.6	32
32	24.0	Male	30→15	AE: herpes zoster	12	16
33	46.0	Male	30→15	AE: acne	12	16
34	37.0	Female	30→15	AE: acne; weight gain	12	16
35	29.0	Female	30→15	Complete remission	10	10
36	28.0	Male	30→15	Complete remission	13	13
37	30.0	Female	30→15	AE: acne	13	16
38	28.0	Male	30→15	AE: weight gain	4	32
39	56.0	Male	30→15	AE: weight gain	10	16
40	27.0	Male	30→15	Complete remission	13	13
41	60.0	Male	30→15	Complete remission	17	17
42	34.0	Female	30→15	AE: herpes zoster	16	16
43	20.0	Female	30→15	AE: herpes simplex	8	16
44	48.0	Female	30→15	AE: nausea	40	16

**Table 4:** Demographic characteristics of the patients treated with upadacitinib in our study. * Baseline EASI was available for 91 patients.

Variable	Value
Patients treated with upadacitinib, n°	165
Male, n (%)	97 (58.8)
Female, n (%)	68 (41.2)
Mean age, years ± SD	41.9 ± 17.1
Mean baseline EASI *	16.44
Initial dose 15 mg, n (%)	93 (56.4)
Initial dose 30 mg, n (%)	72 (43.6)
Patients with ≥1 dose adjustment, n (%)	44 (26.7)
Total dose-adjustment events, n	50
Escalation 15→30 mg, events, n	27
De-escalation 30→15 mg, events, n	23
Patients escalating from 15 mg to 30 mg, n/N (%)	23/93 (24.7)
Median time to escalation, weeks (IQR)	33.1 (19.8–78.5)
Main reason for escalation	Incomplete disease control
Patients reducing from 30 mg to 15 mg, n/N (%)	21/72 (29.2%)
Median time to de-escalation, weeks (IQR)	44.3 (23.1–92.7)
Dose reductions due to adverse events, n	12
Dose reductions due to optimal disease control, n	9
Patients with 30→15→30 pattern, n	4
Patients with 15→30→15 pattern, n	2

**Table 5:** Baseline characteristics according to dose-adjustment status.

Characteristic	Abrocitinib Dose-Adjusted n = 6	Abrocitinib Non-Adjusted n = 41	Upadacitinib Dose-Adjusted n = 44	Upadacitinib Non-Adjusted n = 121
Age, years, mean ± SD	37.0 ± 17.4	39.9 ± 16.1	38.1 ± 13.4	43.3 ± 18.1
Male sex, n (%)	3 (50.0)	20 (48.8)	26 (59.1)	71 (58.7)
Female sex, n (%)	3 (50.0)	21 (51.2)	18 (40.9)	50 (41.3)
Baseline IGA, mean ± SD	3.0	3.0 ± 0.9	2.6 ± 1.0	3.0 ± 1.3
Baseline EASI, mean ± SD *	20.0, n = 1	21.2 ± 6.2, n = 26	15.9 ± 8.7, n = 24	18.2 ± 9.6, n = 67

* EASI at baseline was available for 1 patient who underwent dose adjustment of abrocitinib, 26 patients without dose adjustment of abrocitinib, 24 patients who underwent dose adjustment of upadacitinib, and 67 patients without dose adjustment of upadacitinib.

## Data Availability

Data available on request due to privacy/ethical restrictions.
